# A Protracted Course of Periorbital Oedema in Infectious Mononucleosis Caused by Epstein–Barr Virus

**DOI:** 10.3390/idr14060092

**Published:** 2022-11-23

**Authors:** Daryl Ricardo

**Affiliations:** Department of Emergency Medicine, St. George’s University Hospitals NHS Foundation Trust, Blackshaw Road, London SW17 0QT, UK; daryl.ricardo@doctors.org.uk

**Keywords:** infectious mononucleosis, Epstein–Barr virus, periorbital oedema, Hoagland sign

## Abstract

Infectious mononucleosis (IM) is a viral disease most commonly caused by the Epstein–Barr virus (EBV). The triad of fever, pharyngitis, and cervical chain lymphadenopathy classically describe this benign condition. Ocular association is also possible, although less commonly reported, and manifests as bilateral periorbital oedema presenting early in the disease process. A case of a fit and well 18-year-old female patient who developed periorbital oedema before the classic triad is presented. Furthermore, her case describes a significantly longer duration of periorbital tissue involvement, contrary to what is described in the current literature. Clinicians should not only recognise periorbital oedema as an initial manifestation of IM but also be alerted of its possible protracted course.

## 1. Introduction

Epstein–Barr virus (EBV) is a common pathogen that can cause a variety of systemic infections. Among them is infectious mononucleosis (IM), otherwise known as glandular fever, and presents symptomatically with fever, pharyngitis, neck lymphadenopathy, fatiguability, and splenomegaly [1]. A less clinically ubiquitous sign is bilateral periorbital oedema or ‘Hoagland sign’ [2]. Hoagland sign, presenting either at disease onset or during its course, only lasts for a few days to a few weeks [3,4,5]. Persistence beyond this time frame has not been previously reported in the literature.

This report explores a case of IM with the Hoagland sign as the initial manifestation highlighting its unusually protracted course.

## 2. Case

An 18-year-old female patient was referred by her GP to the emergency department after feeling generally unwell with malaise, fever, sore throat, and puffy eyelids. Upon further questioning, her symptomatology began 10 days prior, initially with generalised fatigue and swollen eyelids (without preceding eyesore), followed by fever, sore throat, and enlarged lymph nodes in her neck. She had no known chronic conditions and no regular medications or allergies. Her observations were as follows: blood pressure 116/70 mmHg, pulse 100 beats per minute, temperature 37.6 ${}^{\circ }$C, respiratory rate 18 breaths per minute, and oxygen saturation 97% on room air.

**Figure 1:**
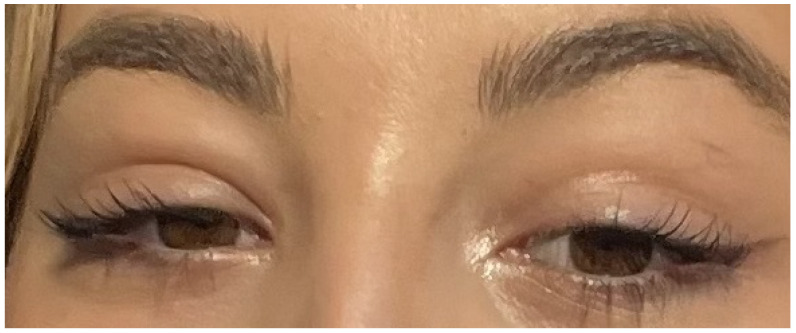
Hoagland sign in our patient on the day of presentation (day 10 of symptoms).

A physical examination revealed non-exudative bilateral tonsillitis, massive bilateral posterior chain lymphadenopathy, a small lymph node in the right supraclavicular fossa, and bilateral periorbital oedema (Figure 1). Interestingly, the oedema was without associated tenderness, erythema, global pain, visual acuity changes, or any other signs suggestive of infection. Although tenderness in her peripheral abdomen was elicited, there was no hepatomegaly or splenomegaly evident on palpation. Neurological, dermatological, and thyroid examinations were normal.

Marked lymphocyte predominant leukocytosis (15.5 × 10${}^{9}$/L, 64.5% atypical lymphocytes) was noted on the haematology panel. C-reactive protein was only mildly elevated (15 mg/L). Mild serum transaminitis (alanine aminotransferase of 88 U/L) was apparent in biochemistry; however, her albumin, renal function, and bone profile were within normal parameters. Upon clinical suspicion of IM, a monospot test was performed that confirmed the presence of heterophile antibodies. For completeness, a urine dipstick test was performed, which showed the absence of proteinuria and haematuria.

She received supportive treatment with intravenous fluids and paracetamol with which her pulse rate normalised and her fever abated. Moreover, she maintained oral intake and did not exhibit any signs of respiratory compromise. She intermittently practised kickboxing as a hobby; hence, advice was given to refrain from the activity or any other contact sport to minimise the occurrence of splenic rupture, a rare but life-threatening complication of IM. Upon discharge, she agreed to take weekly photographs of her eyes to monitor the swelling.

**Figure 2:**
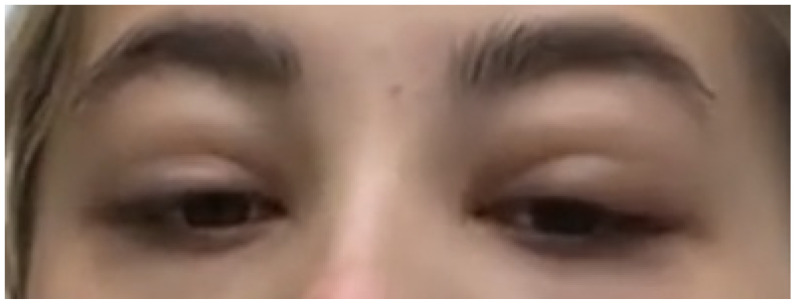
Worsening of oedema during the third week.

**Figure 3:**
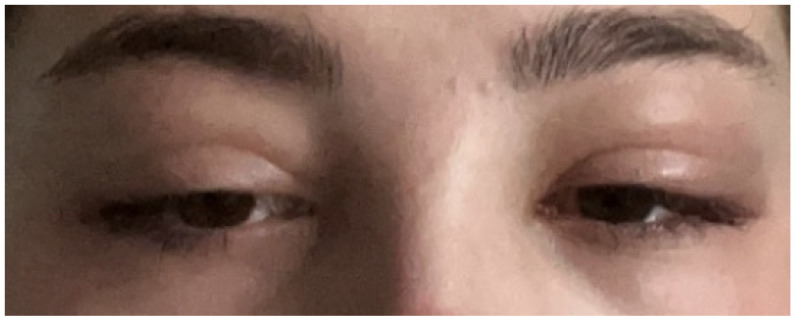
Oedema during the fifth week.

**Figure 4:**
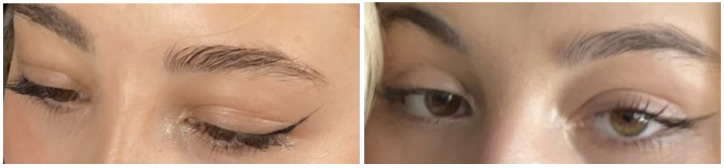
Improvement of oedema began during the sixth week (**left**) and complete resolution occurred in the eighth week (**right**).

In her twelve-week virtual follow-up, she reported that while her fever, sore throat, and neck swelling had subsided after four weeks of symptom onset, the swelling had persisted and reached its worst stage between the third and fifth weeks (Figure 2 and Figure 3). She began to notice improvements in the swelling during the sixth week, and she felt completely at baseline two weeks later (Figure 4). Reassuringly, despite this slow recovery, she did not develop any ophthalmic sequelae or any of the common complications of IM. 

## 3. Discussion

Infectious mononucleosis (IM) is a self-limiting systemic condition primarily caused by EBV. Symptoms such as fever, pharyngitis, and cervical chain lymphadenopathy are most commonly observed [6]. Bilateral periorbital oedema (Hoagland sign) may also be present and can either predate other symptoms or manifest early during the disease process. It first gained recognition in the 1950s when it was noted in up to one-third of IM cases [7]; however, this incidence is not representative and is much lower in standard clinical practice [3].

The pathophysiology of ocular involvement in IM is not yet definitive. HM Bass hypothesised that blocked lymphatic drainage could account for oedema based on the observation that reactive cervical lymphadenopathy also frequently results in glandular oedema, with the caveat that the latter is associated with inflammation but the former is not [8]. A case reported by Süer et al. [9] demonstrated isolated subcutaneous oedema on magnetic resonance imaging. On the contrary, radiological evidence of lacrimal gland inflammation (dacryoadenitis) was described by Burger et al. [10] and Aburn et al. [11] and corroborates the supposed gravitation of the virus towards lymphoid tissues resulting in leukocyte proliferation.

Hoagland sign, if present, is usually transient. Without treatment, oedema subsides within a few days of onset [3,4,12]. Some case reports recorded slightly longer durations of up to two weeks before the full resolution was achieved [2,5,9,13,14]. Where airway patency is threatened, the mainstay of treatment is systemic corticosteroid [15], which has also been shown to result in the rapid dampening of oedema, although its effect remains subjective [10,11]. In our untreated patient, the classical symptoms lasted for four weeks. Interestingly, the complete disappearance of her periorbital oedema did not occur until after eight weeks. To the best of our knowledge, this is the first case report of Hoagland sign taking a protracted course that is significantly beyond the time span reported in the current literature.

Other potential etiologies of bilateral periorbital oedema include periorbital cellulitis, angioedema, cytomegalovirus (CMV) infection, and nephrotic and nephritic syndromes [16,17,18,19]. Absence of other cardinal signs of inflammation (pain, erythema, and warmth) and paucity of atopic features made preseptal cellulitis and angioedema unlikely. While CMV infection may produce mononucleosis-like symptoms [20], it was excluded on the basis of a positive heterophile antibody test. Lastly, normal albumin and negative results for protein and blood on the urine dip made nephrotic and nephritic syndromes incompatible.

## 4. Conclusions

Hoagland sign is an uncommon but important clinical feature that should prompt clinicians to consider IM as a diagnosis. It classically presents early in the disease process and may last between a few days to a few weeks. This case highlights a rather protracted course of recovery and adds value to further defining the natural history of IM, especially where ocular tissue is involved.

## Data Availability

Data are available on request from the author.
